# Scans for Signatures of Selection in Genomes of Wagyu and Buryat Cattle Breeds Reveal Candidate Genes and Genetic Variants for Adaptive Phenotypes and Production Traits

**DOI:** 10.3390/ani14142059

**Published:** 2024-07-13

**Authors:** Alexander V. Igoshin, Grigorii A. Romashov, Andrey A. Yurchenko, Nikolay S. Yudin, Denis M. Larkin

**Affiliations:** 1 The Federal Research Center Institute of Cytology and Genetics, The Siberian Branch of the Russian Academy of Sciences (ICG SB RAS), Novosibirsk 630090, Russia; igoshin@bionet.nsc.ru (A.V.I.);; 2 INSERM U981, Gustave Roussy Cancer Campus, Université Paris Saclay, 94800 Villejuif, France; 3 Royal Veterinary College, University of London, London NW1 0TU, UK

**Keywords:** Wagyu, Buryat cattle, whole-genome resequencing, selection signatures

## Abstract

**Simple Summary:**

Turano-Mongolian cattle breeds are known for adaptation to extreme environmental conditions and for outstanding production traits. The Buryat and Wagyu Turano-Mongolian breeds are characterised by adaptation to harsh climates and poor forage, and top-quality marbled meat, respectively. In our study, we explored the genomes of these breeds to learn about the selection underlying these traits. A set of scans for genetic signatures of selection using complementary approaches allowed us to reveal candidate genes and variants likely shaping the biology of Buryat and Wagyu breeds. These findings could help with livestock improvement efforts.

**Abstract:**

Past and ongoing selection shapes the genomes of livestock breeds. Identifying such signatures of selection allows for uncovering the genetic bases of affected phenotypes, including economically important traits and environmental adaptations, for the further improvement of breed genetics to respond to climate and economic challenges. Turano-Mongolian cattle are a group of taurine breeds known for their adaptation to extreme environmental conditions and outstanding production performance. Buryat Turano-Mongolian cattle are among the few breeds adapted to cold climates and poor forage. Wagyu, on the other hand, is famous for high productivity and unique top-quality marbled meat. We used hapFLK, the de-correlated composite of multiple signals (DCMS), PBS, and F*_ST_* methods to search for signatures of selection in their genomes. The scans revealed signals in genes related to cold adaptation (e.g., *STAT3*, *DOCK5*, *GSTM3*, and *CXCL8*) and food digestibility (*SI*) in the Buryat breed, and growth and development traits (e.g., *RBFOX2* and *SHOX2*) and marbling (e.g., *DGAT1*, *IQGAP2*, *RSRC1*, and *DIP2B*) in Wagyu. Several putatively selected genes associated with reproduction, immunity, and resistance to pathogens were found in both breed genomes. The results of our work could be used for creating new productive adapted breeds or improving the extant breeds.

## 1. Introduction

In livestock, genomic signatures of past and ongoing selection allow for uncovering the genetic bases of economically important traits and environmental adaptations [1]. One of the intensively studied domesticated animals, cattle, is represented by the taurine (*Bos taurus taurus*) and the indicine subspecies (*Bos taurus indicus*). Taurine cattle, in turn, are subdivided mainly into three morphologically and genetically distinct breed groups: European, African, and Turano-Mongolian cattle. Turano-Mongolian cattle are known for adapting to extreme environmental conditions and resisting pathogens [2]. There has been a growing interest in studying the genomes of breeds from this group because of their unique adaptations to harsh climates and excellent production trait phenotypes [3,4,5,6,7,8]. Considering the ongoing issues with climate change and overpopulation, these traits must be genetically dissected.

Until several years ago, Turano-Mongolian cattle in Russian animal husbandry were mainly represented by Kalmyk and Yakut breeds. They have been continuously bred in Russia for centuries and are well adapted to local climatic conditions, including cold winters [8,9]. Until the early 1920s, another hardy native Turano-Mongolian breed called Buryat cattle was present in Siberia. During the Soviet period, they became extinct in Russia, but a herd was saved in neighbouring Mongolia, from where they were reintroduced to Buryatia in the mid-2010s [10,11]. Buryat cattle are a dual-purpose breed that are still well adapted to local climates. The breed is known for its ability to graze year-round in an outdoor pasture system, maintaining viability even with low-caloric and deteriorating forage, resulting in low management costs [12,13]. Several years ago, Wagyu, or Japanese Black, a Turano-Mongolian beef breed originating from Japan, was imported to Russia from the USA and Australia [14]. This commercial Turano-Mongolian breed is known for high productivity and top-quality marbled meat [15].

Owing to their unique features acquired through generations of selective breeding and environmental adaptation, Wagyu and Buryat cattle represent an excellent object for studies on signatures of selection. We used DNA microarray data to study several Russian local breeds, including Buryat cattle [16]. We found several genes putatively selected in this breed. However, the search for signatures of selection based on DNA arrays has at least two major limitations. First, a high density of markers is required to achieve substantial statistical power [17]. Second, genetic markers from arrays in most cases are not causative, but linked markers, which reduces their potential use in marker-assisted selection [18]. The use of whole-genome resequencing (WGR) data is not limited in the same way. For example, previous studies based on WGR identified a few likely causative or unique genetic variants in cattle (e.g., [8,19,20,21]). In Wagyu, signatures of selection have been previously explored using DNA microarray and WGR data [5,22]. This resulted in multiple candidate genes for marbling and other meat traits. Yet, very few to no candidate DNA polymorphisms were reported in these studies.

Herein, we conduct a comprehensive study to identify signatures of selection in Russian Wagyu and Buryat cattle populations using WGR data and a range of complementary methods. We report candidate variants likely underlying observed selection signals and that, therefore, are involved in shaping the biology of the breeds under study. In our research, the hypothesis was that genomes of commercial Wagyu cattle contain multiple signatures of selection in genes related to production phenotypes. In contrast, native Buryat cattle genomes preferably contain selection signals in genes related to environmental adaptations.

## 2. Materials and Methods

### 2.1. Data Preparation

DNA samples of Wagyu (*n* = 20), Buryat (*n* = 20), additional Turano-Mongolian Hanwoo (*n* = 20) and Yakut (*n* = 20) breeds, and European taurine Kholmogory (*n* = 20) cattle were used. The Turano-Mongolian Hanwoo and Yakut breeds were chosen for the signatures of selection scans being the closest breeds to Buryat and Wagyu according to previous population structure analyses, haplotype sharing, and phylogeny [10]. Kholmogory, a breed of European origin, was used as a non-Turano-Mongolian outgroup. Resequencing data for Hanwoo, Yakut, and Kholmogory breeds were downloaded from the NCBI database (see Table S1 for SRA IDs). The Wagyu and Buryat samples underwent resequencing using the Hiseq4000 platform at Novogene Co., Ltd. (Beijing, China) for ~50 Gbp (~15× coverage). The raw resequencing data were processed following the 1000 Bull Genomes Project (1KBGP) guidelines [23]. Briefly, reads were cleaned with Trimmomatic v.0.38 [24] in PE mode using relevant Illumina adapters (File S1) and with the following parameters: LEADING:20 TRAILING:20 SLIDINGWINDOW:3:15 AVGQUAL:20 MINLEN:35. The cleaned reads were mapped to the reference cattle genome (ARS-UCD1.2_Btau5.0.1Y) using BWA-MEM v.0.7.17 [25], and then duplicate reads were marked with Picard v.2.18.2 [26] with the parameter OPTICAL_DUPLICATE_PIXEL_DISTANCE=”2500” to match the sequencing technology. Next, we performed a base quality score recalibration (based on the ARS1.2PlusY_BQSR_v3 dataset) and a follow-up variant calling procedure using GATK v.3.8-1-0-gf15c1c3ef [27]. The resulting Buryat and Wagyu gVCF files were shared with the 1KBGP (Run 9).

We used the hapFLK, de-correlated composite of multiple signals (DCMS), population branch statistic (PBS), and F*_ST_* (fixation index) methods to search for signatures of selection in the Wagyu and Buryat genomes. For the signatures of selection analysis using the hapFLK, DCMS, and PBS methods, two datasets were generated, each including the genomes of one breed of interest (Wagyu/Buryat), Hanwoo, Yakut, and Kholmogory. The gVCF files were merged using the GenotypeGVCFs subroutine of the GATK package. The resulting VCF files contained 45,325,282 and 34,128,857 polymorphic sites (single-nucleotide polymorphisms and indels) in the Buryat and Wagyu sets, respectively.

Only single-nucleotide polymorphisms (SNPs) were used in further analysis, which were additionally filtered using hard-filtering parameters (“QD < 2.0|| FS > 60.000|| MQ < 40.00|| MQRankSum < −12.5|| ReadPosRankSum < −8.0”) by the VariantFiltration GATK subroutine, according to GATK’s best-practice recommendations [28]. The multiallelic SNPs were split and the resulting datasets were used for the DCMS and PBS analyses. For the hapFLK analysis, multiallelic SNPs were removed, and the datasets were filtered for minor allele frequency (MAF) < 10% and a fraction of the successfully genotyped individuals (*--maf 0.1 --geno 0.1*) in PLINK (v. 2.00) [29]. After these filtering steps, the Buryat and Wagyu datasets contained 10,308,949 and 10,276,234 SNPs, respectively.

For the F*_ST_* scans, the VCF files of the taurine 1KBGP (Run 9) dataset were used. Indels were removed using the “-T SelectVariants -selectType SNP” options in GATK (v. 3.8-1-0-gf15c1c3ef), and multiallelic SNPs were further split in biallelic SNPs using BCFtools version 1.11 (“norm -m -any” option) [30]. Data obtained at this stage were utilised for the window-based F*_ST_* scan. Next, the whole dataset was filtered for a fraction of the successfully genotyped individuals (BCFtools: F_MISSING < 0.1). After creating a separate dataset for each breed, only individuals with at least 80% successful genotyping and an alternative allele frequency of at least 10% were included for the target breeds (i.e., Wagyu/Buryat). The datasets used for the single-point F*_ST_* scan were the Buryat and Wagyu datasets, containing 12,353,594 and 10,410,733 SNPs, respectively.

### 2.2. Selection Signatures Scans

#### 2.2.1. HapFLK

The hapFLK test allows for the detection of selected genome intervals based on differences in haplotype frequencies between populations, considering their hierarchical structure [31]. The number of haplotype clusters (K) was estimated for the Buryat and Wagyu datasets as 15 and 20, respectively, using fastPhase software (v. 1.2) [32]. HapFLK v.1.4 software was used for analysis with the following parameters: “*-K 15\20 --nfit = 30 -kfrq*”. *p*-values were calculated using normal distribution as a null model with the “*MASS*” R package (“*rlm*” function) [33]. The q-values were then calculated using the “*qvalue*” R package [34]. Statistically significant intervals were defined by at least one SNP with a q-value < 0.01, and interval boundaries were set by the first SNPs with q-values > 0.2 upstream and downstream of significant SNPs. For these intervals, haplotype diversity (using the *hapflk-clusterplot.R* script) and local trees (using *local_reynolds.py* and *local_trees.R* scripts) were visualised to find the breed under selection.

#### 2.2.2. DCMS

The DCMS statistic allows us to combine the results of several tests into a single summary statistic, considering the correlation between them, thus increasing the statistical power of analysis [17]. In our DCMS framework, we used H1, H12 [35], iSAFE [36], XPnSL [37], and T [38] statistics. Before testing, the VCF files were phased using Shapeit4.2 [39], assuming an effective population size of 1000 with the MCMC algorithm parameter “*--mcmc-iterations 10b,1p,1b,1p,1b,1p,1b,1p,10m*”. A genetic map from Qanbari et al., 2020 [40] (likelihood-based version), was used for the recombination rates between SNPs.

The H1 statistic applies the expected homozygosity formula for the multiallelic locus to fixed-length haplotypes. The H12 statistic is based on the same principle, but its calculation combines the frequencies of the two most common haplotypes, allowing for a better detection of “soft sweeps” [35]. The *H12_H2H1.py* script (https://github.com/ngarud/SelectionHapStats/blob/master/scripts/H12_H2H1.py (accessed on 6 August 2021)) was used to calculate H1 and H12 statistics. The window size (-w), step (-j), and the allowed number of mismatched alleles (-d) were chosen to be 200, 10, and 4, respectively.

The iSAFE method allows for the mapping of causative variants underlying signatures of selection with high accuracy based on the coalescent theory [36]. For our analysis, we used the *isafe.py* script version 1.0.7 (https://github.com/alek0991/iSAFE/releases/tag/v1.0.7 (accessed on 6 August 2021)). Calculations were performed in 3 Mbp windows with 1 Mbp increments. Statistics for all 3 Mbp intervals were combined into a single file, discarding the first and last megabases in each window.

The XP-nSL method detects signatures of selection by comparing haplotype diversity between the case and control populations. This approach has good statistical power under various adaptive allele selection scenarios. The selscan software (v. 1.3.0) [37] was used for the analysis. Yakut, Hanwoo, and Kholmogory breeds were used as controls.

The T-statistic is a likelihood ratio test comparing the neutral and selection models based on the observed haplotype frequency spectra. This statistic shows high power for different adaptive allele selection models and demographic scenarios, but may inflate when analysing admixed populations and regions of the genome with low recombination rates [38]. Lassip software (v. 1.1.0) [41] was used to calculate T-statistics. First, haplotype frequency spectra were calculated (options: *--calc-spec --hapstats --lassi*) in windows of 200 SNPs and steps of 10 SNPs. Then, T-statistic values were obtained for the windows (options: *--spectra --lassi*).

The files with the statistics obtained using the five methods described above were translated into bed format and combined into a single file using bedtools (command: closest) [42]. The numbers of SNPs were 2,192,773 and 1,190,487 for the Buryat and Wagyu breeds, respectively.

Covariance matrices for the five statistics were calculated using the “*CovNAMcd*” function of the “*rrcovNA*” R-package [43] with parameters alpha = 0.75 (fraction of sampled SNPs) and nsamp = 500,000 (number of samples). The DCMS statistic values were obtained for each SNP using the “*DCMS*” function of the “*MINOTAUR*” R-package [44]. The *p*-values, q-values, and interval boundaries for the selected intervals were calculated as described for hapFLK.

#### 2.2.3. F_ST_

SNPs and genome regions demonstrating divergence between the target breeds and the global cattle population may contribute to breed-specific phenotypes. To identify such SNPs and genome intervals, we used data from the taurine set of the 1KBGP (Run 9) to calculate the F*_ST_* statistics between the Wagyu and Buryat breeds, on the one hand, and other global breeds, on the other. VCFtools software (v.0.1.13) [45] was used for the calculation with the “*--fst-window-size 50000 --fst-window-step 25000 -max-missing 0.9*” options for the window-based analysis, and without the “*--fst-window-size, --fst-window-step*” options for individual SNPs. Single-point F*_ST_* analysis was performed for SNPs with MAF > 0.1 and at least 80% successfully genotyped animals in the target breed. The fraction of genotyped animals per SNP in the 1KBGP taurine subset was set to >90%. Based on the results of the window-based analysis, 0.1% of the top-weighted F*_ST_* values were chosen. From the single-point analysis, SNPs with top 1% F*_ST_* values were annotated.

#### 2.2.4. PBS

Population Branch Statistics (PBS) allows the estimation of population-specific allele frequency shifts based on pairwise F*_ST_* values among several populations [46,47]. For the PBS analysis, we calculated weighted F*_ST_* values in 70 kb windows with 35 Kb steps for all breed pairs using VCFtools v.0.1.13 [45]. Only windows with >200 SNPs were used in the analysis. The weighted F*_ST_* values were transformed into units of scaled time *T = − log(1 − F_ST_)*, and then placed into an equation for four population-based PBS calculations [46,48]. In the PBS calculations, Yakut and Hanwoo were considered closest to the Buryat and Wagyu breeds, respectively, as suggested by population structure, haplotype sharing, and phylogeny [10]. As candidate regions, the top 0.1% of PBS windows were considered.

### 2.3. SNP Annotation, Search for Candidate Causative Variants, and Enrichment Analysis

SNPs identified in the single-point F*_ST_* analysis, and detected by any of the four window-based methods, were annotated using the SNPeff tool [49]. For genes with no known function, a search for homologs was conducted using the BlastP tool [50]. To search for candidate causative variants within the putatively selected regions, we calculated alternative allele frequencies for all SNPs located wherein, except those annotated as intergenic or intronic. The calculations were carried out for a respective four-breed dataset (i.e., Wagyu/Buryat + Hanwoo + Yakut + Kholmogory) and taurine breeds of the 1KBGP.

Because the computation of the hapFLK, PBS, and DCMS statistics involved three additional breeds (Yakut, Hanwoo, and Kholmogory), as candidate causative variants for regions detected by these methods, we considered those having ≥ 0.3 difference in alternative allele frequency between a target breed and the abovementioned control breeds. This threshold has been used to include variants in multiple-mutation soft sweeps [51]. For regions identified by the window-based F*_ST_* method, we considered SNPs with a ≥ 0.3 difference in alternative allele frequency between a target breed and the rest of the 1KBGP as candidate causative variants. Where needed, as supporting information for the haplotype diversity plots, a single-point F*_ST_* was calculated between a target breed and the three control breeds.

The DAVID tool [52] was used for functional enrichment analysis and functional annotation clustering. Both types of analysis were performed for each of the two breeds. For each breed, in turn, two gene sets were analysed. The first one was composed of genes containing variations with the highest potential effect (missense and nonsense substitutions, start-and-stop codon losses, and mutations in the acceptor and donor splicing sites), resulting from the single-point F*_ST_* analysis (top 1%). The second set included all genes with overlapping candidate regions detected by at least one method. The background list for the DAVID tool was compiled using the BioMart tool [53] based on the Ensembl Genes 108 database. The q-value (FDR) of 0.05 was the threshold for significance in the functional enrichment analysis. An enrichment score (ES) of >1.3 was used for the functional annotation clustering.

## 3. Results

### 3.1. Statistics of Window-Based and Single-Point Scans for Selection Signatures

**Figure 1:**
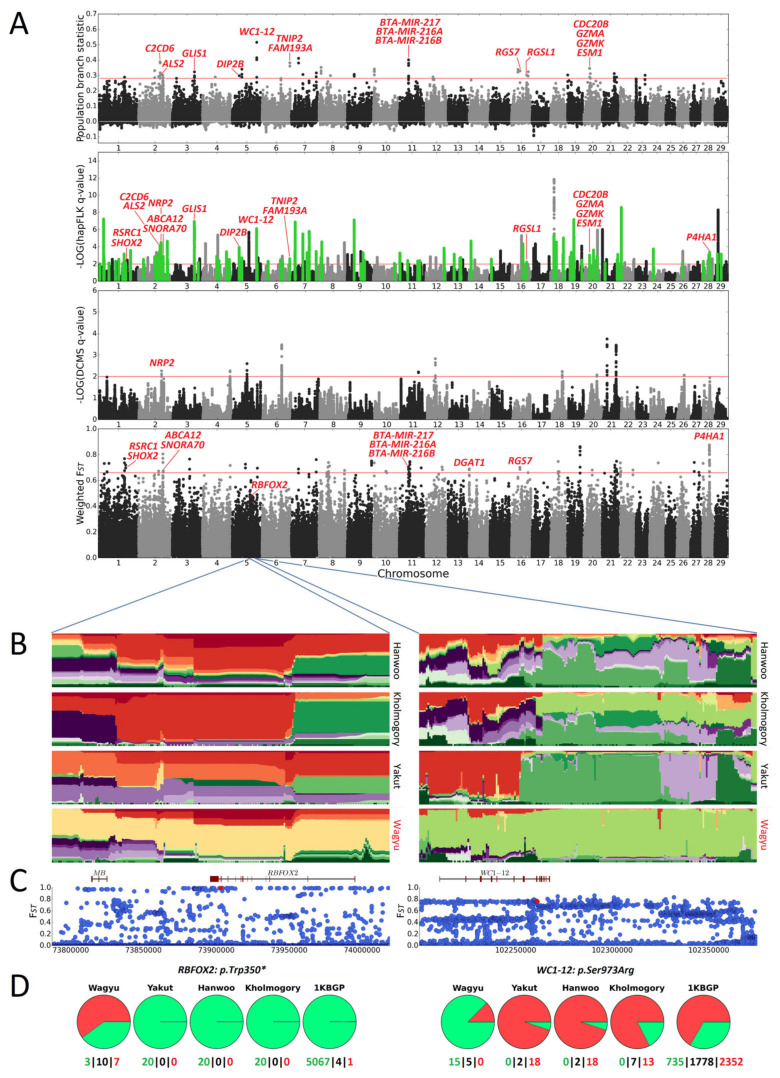
(**A**) Manhattan plots for PBS, hapFLK, DCMS, and window-based F*_ST_* analyses for the Wagyu breed. Red horizontal lines indicate a significance threshold of q = 0.01 (hapFLK, DCMS) or 0.1% cutoff (PBS, window-based F*_ST_*). In the plot for hapFLK statistics, regions putatively selected in the Wagyu cattle are in green. (**B**,**C**) Haplotype diversity plots. (**B**) Single-point F*_ST_* values (**C**) for *RBFOX2* and *WC1-12* gene regions. F*_ST_* is calculated between Wagyu, on the one hand, and either 1KBGP (*RBFOX2* region) or the three other breeds (*WC1-12* region) on the other. Red dots indicate candidate variants. (**D**) Pie charts depicting allele frequencies in breeds/groups for the respective candidate variants. The green and red colours show the reference and alternative alleles, respectively. At the bottom of the pie charts, the numbers of homozygous and heterozygous individuals are indicated.

A complete list of regions detected in the Wagyu and Buryat breeds is presented in Table S2. The hapFLK analysis performed for the Wagyu dataset detected 181 putatively selected genomic intervals (Figure 1), of which 99 regions containing 276 genes were assigned to Wagyu. The DCMS method identified 13 candidate regions (17 genes) in this breed. In addition, 63 intervals identified with PBS were consolidated into 36 non-overlapping regions (37 genes). From the window-based F_ST_ analysis, the top 99 intervals were chosen and combined into 51 non-overlapping regions (57 genes). In total, 33 genomic regions were detected in Wagyu by >1 method. Of them, 18 contained genes. The candidate SNPs for the regions revealed in this breed are shown in Table S3.

**Figure 2:**
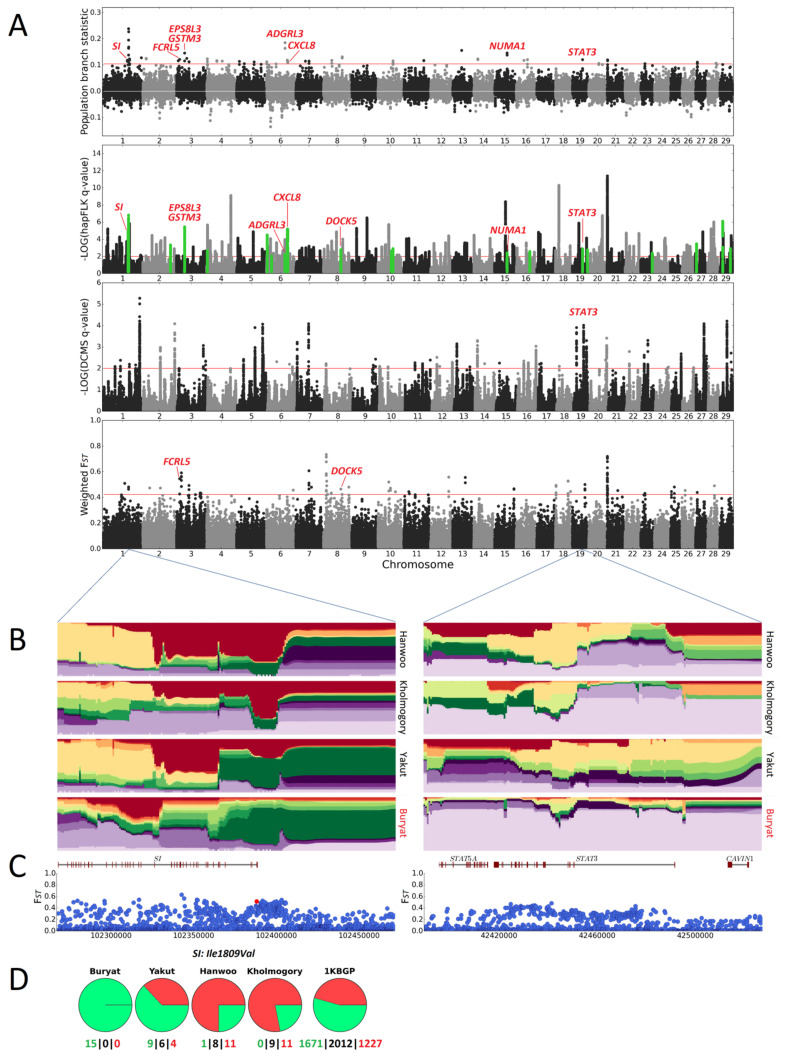
(**A**) Manhattan plots for PBS, hapFLK, DCMS, and window-based F*_ST_* analyses of Buryat cattle. Red horizontal lines indicate a significance threshold of q = 0.01 (hapFLK, DCMS) or 0.1% cutoff (PBS, window-based F*_ST_*). In the plot for hapFLK statistics, regions putatively selected in the Buryat cattle are in green. (**B**,**C**) Haplotype diversity plots. (**B**) Single-point F*_ST_* values (**C**) for *SI* and *STAT3* gene regions. F*_ST_* is calculated between Buryat and the three other breeds. A red dot indicates a candidate variant. (**D**) Pie charts depicting allele frequencies in breeds/groups for the respective candidate variant. The green and red colours show the reference and alternative alleles, respectively. At the bottom of the pie charts, the numbers of homozygous and heterozygous individuals are shown for each allele.

**Figure 3:**
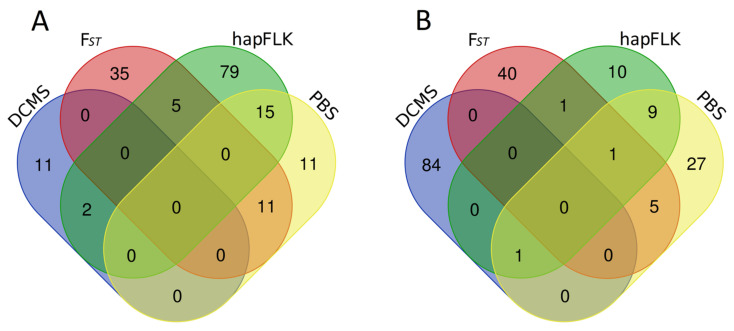
Venn diagrams illustrating the intersection statistics between window-based methods for (**A**) Wagyu and (**B**) Buryat breeds.

The hapFLK analysis on the Buryat cattle dataset resulted in 293 intervals (see Figure 2). Of those, 22 regions (28 genes) were under putative selection in the Buryat breed. The DCMS analysis detected 85 regions containing 93 genes. The PBS and window-based F_ST_ analyses led to 68 and 99 intervals, further combined into 42 (38 genes) and 47 (59 genes) non-overlapping regions, respectively. Overall, 17 candidate regions containing eight genes were detected in the Buryat cattle with >1 method. The candidate SNPs for the regions revealed in this breed are shown in Table S4. According to the literature, most genes within the regions detected in the Wagyu and Buryat breeds and supported by the >1 method could be related to adaptive and productive traits (Table 1). The intersection between the different window-based methods for the Wagyu and Buryat breeds is illustrated in Figure 3.

In the top 1% of results of the single-point F_ST_ analysis of Wagyu cattle, 464 missense variants, 10 nonsense variants, 4 splice donor variants, 4 splice acceptor variants, 1 start loss and 1 stop loss were present (Table S5). In the Buryat cattle in the same analysis, 337 missense variants, 11 nonsense variants, and 2 splice acceptor variants were identified (Table S6).

### 3.2. Candidate Genes and Variants

#### 3.2.1. Cold Climate Adaptation

The *STAT3* (signal transducer and activator of transcription 3) gene is mostly found in the consensus region BTA19:42.42–42.49 Mbp, and was detected by three methods in the Buryat breed (Figure 2B,C). *STAT3* encodes for a member of the STAT family of transcription factors, which are mainly involved in cytokine signalling. *STAT3* plays an important role in thermoregulation [58,59,60]. According to Gao et al. in 2004, the knockout of *STAT3* in the central nervous system in mice results in the inability of animals to maintain their body temperature during cold challenge at 4 °C [61]. The BTA19:42.42–42.49 Mbp region lacks any variants with a potentially high functional impact and high F*_ST_*; putative selection might have acted on haplotypes or indels rather than single-nucleotide variants.

Another top candidate for cold climate adaptation in Buryat cattle is the *DOCK5* (dedicator of cytokinesis 5) gene harbouring selection signal (BTA8:72.60–72.65 Mbp), as supported by hapFLK and F*_ST_*. *DOCK5* encodes a member of the dedicator of cytokinesis protein family. According to a study by Lai and colleagues (2020), *DOCK5* regulates energy balance and insulin sensitivity [57]. Moreover, *DOCK5*^−/−^ mice demonstrate lower body temperature when compared to control animals. As for *STAT3*, we did not detect candidate variants for the *DOCK5* gene within the BTA8:72.60–72.65 Mbp region.

#### 3.2.2. Growth and Development

The highest F*_ST_* between the Wagyu and 1KBGP datasets was observed for the nonsense variant (BTA5:73903010, p.Trp350*, F*_ST_* = 0.992) in one of the isoforms (*ENSBTAT00000073356.1*) of the *RBFOX2* (RNA binding Fox-1 homolog 2) gene (Figure 1B–D). This variant has a frequency of 0.6 in Wagyu and 0.0006 in the 1KBGP set (present in one Buryat individual and four animals of unknown breed). The *RBFOX2* gene is part of the RBFOX family of highly conserved splicing regulators. These regulators play a crucial role in muscle tissue development and the maintenance of muscle mass in animals [111]. The knockout of *RBFOX1* and *RBFOX2* in mice results in significant muscle atrophy. At the same time, a single knockout of either of these two genes did not cause this effect [112]. Also, according to Júnior et al. [113], the *RBFOX2* gene in Nelore cattle is associated with the ribeye area, an important characteristic of beef breeds [114].

The *SHOX2* (short stature homeobox protein 2) gene is largely overlapped by the BTA1:109.35–109.43 Mbp region at the intersection of two methods in the Wagyu breed. *SHOX2* encodes a member of the homeobox family. This gene participates in bone formation and neural and muscular development [70,71]. In cattle, it was previously connected to longissimus muscle development in the transcriptome analysis of Shandong Black and Luxi breeds [72].

#### 3.2.3. Feed Efficiency Traits and Metabolism

The BTA1:102.34–102.41 Mbp interval in the Buryat breed overlaps with the *SI* gene encoding for sucrase-isomaltase. In ruminants, starch digestibility, and therefore feed efficiency, greatly depends on the activity of this enzyme [65,66]. The most promising candidate SNP for the *SI* gene is the missense variant BTA1:102388029 (p.Ile1809Val). The alternative allele of BTA1:102388029 is not present in the Buryat breed, while in Kholmogory, Hanwoo, Yakut, and the 1KBGP, it has frequencies of 0.78, 0.75, 0.37, and 0.45, respectively. This suggests that, in the Buryat breed, haplotypes containing the alternative allele at BTA1:102388029 were potentially eliminated by selective pressure.

The previously mentioned *SHOX2* gene detected in Wagyu, apart from its role in morphogenesis, is also related to feed efficiency traits in cattle. Thus, in a genome-wide association study, this gene was associated with residual feed intake (RFI) in Angus cattle [68]. In the Kinsella breed, the *SHOX2* mRNA was differentially expressed in rumen tissue between animals with high and low residual feed intake [67].

Wagyu’s missense variant (BTA1:100006396, p.Lys264Met, F_ST_ = 0.989) from the *SERPINI2* (serpin family I member 2) gene was among the top-ranked SNPs. *SERPINI2* encodes a member of the family of proteins acting as inhibitors of serine proteases (https://www.genecards.org/cgi-bin/carddisp.pl?gene=SERPINI2 (accessed on 1 July 2024)). It was shown that, in vitro, the *SERPINI2* product pancpin inhibits pancreatic chymotrypsin and elastase [115]. The *SERPINI2* gene is differentially expressed in the liver of beef steers with high and low average daily weight gain, with the direction of effect differing between breeds [116]. Also, this gene’s mRNA expression in the liver is correlated with the feed conversion ratio in the Angus breed [117]. In Wagyu, the alternative allele of the SNP has a frequency and carrier rate of 0.58 and 0.75, respectively. This allele is also found in Yanbian (frequency of 0.09) cattle and several animals of unknown breed.

#### 3.2.4. Meat Quality Traits

The window-based F_ST_ analysis detected the BTA14:0.58–0.65 Mbp region in Wagyu, with the *DGAT1* (diacylglycerol O-acyltransferase 1) gene in the middle. *DGAT1* encodes a key enzyme in triglyceride synthesis in mammals [118]. This gene contains the dinucleotide change GpC > ApA at the BTA14:611019-611020 site, which leads to the substitution of alanine for lysine at residue 232. In Wagyu, the frequency of the lysine allele reaches 0.88, while in the 1KBGP set, it has a frequency of 0.14. In a study on Swedish beef cattle breeds, heterozygote lysine carriers had an increased marbling score and intramuscular fat content (also directly related to meat marbling) compared to alanine homozygote carriers (lysine homozygotes were absent) [119]. Another study on Hanwoo cattle showed a significantly higher marbling score with lysine homozygotes compared to heterozygotes (alanine homozygotes were not detected); however, this was only in the presence of the T11993C variant [120]. It has been reported that the homozygous genotype for the lysine allele is linked to an increased intramuscular fat content in the German Holstein breed [121].

The *IQGAP2* (IQ motif containing GTPase activating protein 2) gene with a top-ranked missense variant (BTA10:7901963, p.Leu1103Phe, F_ST_ = 0.988) might also be controlling meat traits in Wagyu. This gene encodes Ras GTPase-activating-like protein, which, among other functions, is implicated in hepatic fatty acid uptake regulation and glycogen synthesis [122,123]. A study on Angus steers reported that the expression of *IQGAP2* is negatively correlated with marbling score [124]. According to a genome-wide association study on the Hanwoo breed, *IQGAP2* is associated with total meat collagen content, contributing to meat tenderness and texture [97]. In pigs, this gene is associated with meat tenderness [125]. The alternative allele of the BTA10:7901963 SNP in Wagyu has a frequency and carrier rate of 0.63 and 0.85, respectively. In the 1KBGP, the frequency of this allele is 0.0015 (mainly present in Turano-Mongolian breeds: Hanwoo (0.14), Kazakh (0.06), Menggu (0.05), Altai (0.03), and Buryat (0.03)).

#### 3.2.5. Immunity and Resistance to Pathogens

Apart from its possible role in cold adaptation, the *STAT3* gene detected in Buryat cattle and described above controls inflammation and immunity in vertebrates [88]. In bovine endometrium, the STAT3 signalling pathway plays a role in mucosal innate immunity [89].

At the intersection of two methods, we also found a region overlapping the *WC1-12* (WC1 isolate DV10) gene in Wagyu, which encodes a member of the WC1 co-receptors expressed by γδT cells [90]. This region is likely under selection in Wagyu and Yakut cattle (Figure 1B–D). In cattle, WC1 is involved in the immune response to bovine respiratory disease [91] and Mycobacterium bovis infection (the main cause of bovine tuberculosis) [92]. We found 16 missense variants in *WC1-12* with F*_ST_* > 0.4 between Wagyu, on the one hand, and Hanwoo, Kholmogory, and Yakut on the other (Table S3). Of them, the most promising candidate variant is BTA5:102260891 (p.Ser973Arg), having the highest differentiation (F*_ST_* = 0.77) between Wagyu and the other breeds (Figure 1D).

#### 3.2.6. Reproduction

The second-top SNP from the single-point F*_ST_* analysis in Wagyu is a missense variant (BTA10:3638331, p.Ala21Pro, F*_ST_* = 0.991) in the *ENSBTAG00000023186* gene. The alternative allele of this SNP has a frequency of 0.78, so all animals are either homozygous or heterozygous carriers. In contrast, in the 1KBGP, this variant is rare with a frequency of 0.0022, segregating in four Turano-Mongolian breeds and several animals of unknown breed, with the highest frequency in Japanese Native cattle (0.44). The *ENSBTAG00000023186* gene is one of the top hub genes in the gene module differentially expressed between high- and low-fertile cows [126]. The BlastP search revealed that *ENSBTAG00000023186* has a 99.2% amino acid sequence identity with the bovine *MRPS17* gene located on BTA25, with 100% coverage. The *MRPS17* gene encodes mitochondrial ribosomal protein S17, likely participates in the mitochondrial translation system (https://www.genecards.org/cgi-bin/carddisp.pl?gene=MRPS17 (accessed on 1 July 2024)), and is also associated with reproductive traits in cattle [127].

#### 3.2.7. Milk Production Traits

In the dual-purpose Buryat breed known for an increased milk protein percentage [128], the region of BTA6:77.46–77.56 Mbp overlapping with the *ADGRL3* (adhesion G protein-coupled receptor L3) gene was detected by two methods. *ADGRL3* encodes for a member of the G-protein coupled receptors [129] and plays a significant role in neurophysiological processes such as neuron guidance, signal transduction, and central nervous system development [130]. According to a large-scale genome-wide association study on Holsteins, *ADGRL3* is associated with milk protein yield and percentage [101]. No potential high-impact candidate variants were found for the *ADGRL3* gene within the selected region.

### 3.3. Functional Enrichment Analysis and Functional Annotation Clustering

In Wagyu cattle, the functional enrichment analysis carried out for genes detected by window-based methods resulted in 30 statistically significant (q < 0.05) terms, mostly related to the metabolism of metal ions and lipoxygenase enzymes (Table S7). This analysis of the genes highlighted by the single-point F_ST_ scan resulted in five terms. In the functional annotation clustering carried out on genes from the window-based scans in this breed, six clusters were enriched (ES > 1.3) (Table S8). Top clusters were related to the metabolism of metal ions (ES = 5.1), the homeobox domain (ES = 3), and lipoxygenase enzymes (ES = 2.8). The same analysis based on a single-point F_ST_ scan resulted in eight enriched clusters, the top ones being linked to epidermal growth factor (ES = 2.3), the transmembrane helix (ES = 2.1), and immunoglobulin (ES = 1.9).

The functional enrichment analysis performed on the genes detected by the window-based methods in the Buryat cattle resulted in a single significant term (“Glutathione S-transferase, Mu class”) (Table S7). This analysis, based on a single-point scan, did not reveal any terms. As a result of the functional annotation clustering, three clusters were enriched, with the top one related to glutathione metabolism (ES = 2.2) (Table S8). The analysis based on a single-point F_ST_ scan resulted in four enriched clusters, the top of which (ES = 2.2) comprised various terms with little in common.

## 4. Discussion

We used several methods to identify specific regions of Wagyu and Buryat cattle genomes that have likely undergone natural or artificial selection. Our work highlights genome regions identified by selection scans and identifies several candidate causative SNPs likely affected by selection. We anticipate these genetic variations will contribute to future advancements in gene editing, marker-assisted selection, and genomic selection. They could help enhance economically important and adaptive traits in these and other cattle breeds.

The results of our work are in good agreement with the features of the Buryat and Wagyu breeds. Thus, the revealed selection signatures and candidate variants in several genes (e.g., *DGAT1* and *IQGAP2*) likely contribute to meat marbling. A nonsense mutation found in a high frequency in Wagyu (but that was nearly absent from the 1000 Bull Genomes Dataset) in a gene related to muscle development (*RBFOX2*) makes this change one of the top candidates for Wagyu meat traits. The enrichment of functional terms linked to lipoxygenase enzymes in the same breed may contribute to the so-called “Wagyu beef aroma”, which has been shown to depend on the activity of lipoxygenases to a great extent [131]. Lipoxygenases in mammals, however, play a role in various conditions, such as cancer, cardiovascular disease, and immune and neurological disorders [132]. The observed enrichment, therefore, may be related to other adaptations, and requires additional investigation.

The terms related to the metabolism of metal ions probably reflect the selection of alleles in genes controlling the intake of trace elements. This is highly relevant for Wagyu since beef marbling depends on their consumption. On the other hand, in the Buryat breed, selection signatures in the *SI* gene might contribute to this breed’s unique ability to exist under low-caloric and deteriorating forage [65,66]. The fact that there are multiple genes related to cold adaptation and disease resistance under selection in this breed is likely to contribute to Buryat cattle formation in Western Siberia, known for its cold climates. Their relatively short migration to Mongolia and back likely did not impact cold adaptation phenotypes because the climates in northern Mongolia and Buryatia are similar [133,134]. The functional enrichment “*Glutathione S-transferase. Mu class*” term could also be related to cold adaptation because glutathione-S-transferase genes are involved in the antioxidant defence system of mammalian cells during prolonged cold stress [135,136,137].

One of the strongest selection signals in Buryat cattle was found in the *STAT3* gene, which shows evidence of contributing to thermoregulation under cold conditions in mammals [58,61]. However, this gene was not selected in other cold-adapted breeds in our study (Yakut and Kholmogory) or other studies (Chinese Yanbian) [8,16,21]. However, in cold-adapted Chinese Xinjiang Brown cattle suffering from bovine respiratory disease, *STAT3* was found to be differentially methylated [138]. Thus, rare convergent selection events at the gene level in environmental adaptations [139] support the need for studying individual breeds residing in different environments.

The selection signatures in several genes possibly related to cold adaptation (*RSRC1* (arginine and serine-rich coiled-coil 1), *RGS7* (regulator of G protein signaling 7), and *RGSL1* (regulator of G protein signaling like 1) in Wagyu have two possible explanations. On the one hand, genes contributing to adaptation to low temperatures often have pleiotropic effects [139,140] and, therefore, could be associated with production traits. On the other hand, ancestral Turano-Mongolian cattle have formed in cold climates of Asia [141], suggesting that Wagyu could inherit some of these adaptations from their ancestors together with other extant Turano-Mongolian cattle breeds.

Overall, Wagyu demonstrates stronger selection signals than Buryat cattle when comparing the magnitude of the signals in the same methods. A similar effect is observed when comparing alternative allele frequencies in the single-point F*_ST_* analysis. Among the top SNPs in Buryat cattle, less than three percent show F*_ST_* > 0.3, while in Wagyu, 60% of the top SNPs have F*_ST_* > 0.3. We believe these differences are related to intensive artificial selection for production traits in Wagyu. Buryat cattle likely escaped intensive artificial selection during their natural adaptation to harsh climates, which were not significantly different from the climates in which the ancestral Turano-Mongolian population had formed.

It is worth mentioning that >90% of candidate genes identified in the present study in Buryat cattle are novel, relative to our previous SNP array study of the same breed [16]. When our Wagyu results are compared to a WGS study by Shi et al., 2023 [5], the same fraction of novel genes are found (>90%). Despite both studies using whole-genome resequencing data, the number of animals in the Shi et al. 2023 paper is smaller for Wagyu (11 animals vs. 20 in our study) and other breeds. The methods are mostly different, apart from the window-based F*_ST_* scan, likely explaining at least in part the difference in results between these studies.

Eleven genes overlap between the SNP array-based and WGS studies of Buryat cattle. Despite none of them being confirmed by the >1 method in the present study, and therefore not being described above, one of them, *CCND2* was earlier associated with body weight in cold-adapted Siberian beef cattle populations [142]. In Wagyu, 29 genes overlap between the present study and the study by Shi et al. in 2023 [5]. From these, *DIP2B* (disco-interacting protein 2 homolog b) is at the intersection of two statistics in our study. The study by Shi et al. from 2023 does not report any candidate genetic variants, nor the top candidate nonsense mutation in *RBFOX2* reported herein.

One limitation of our work is the relatively small sample size for the breeds under study. Despite satisfying the need for proper statistical power in most signatures of selection scans [17], increasing the sample size would likely increase statistical power and reduce the number of false-positive signals. This could also increase signal overlap between methods. In general, however, there is a relatively small expectation that the selection signals detected by all methods will overlap [143,144,145]. Each method has a different underlying hypothesis (e.g., F*_ST_* assumes substantial deviation in allele frequencies between the study and control population [146], while H1 expects a high level of haplotypic homozygosity in a selected region [35]), and finding intervals that satisfy all of them at the same time is hard. The importance of larger sample sizes becomes apparent in the comparison to the study by Shi et al. (2023) mentioned above. Another limitation is the lack of animals from the original Wagyu population from Japan due to export limitations. Animals used herein were descendants of animals imported from the USA and Australia [14], and animals used in the study by Shi et al. in 2023 were from China [5]. A possible confounding factor in this study is that we analysed reintroduced cattle rather than the original Buryat cattle population. However, as indicated by estimates of inbreeding, heterozygosity, and effective population size, the population used for reintroduction likely escaped a strong bottleneck effect [10]. This means that the genetic profile of the studied population is likely not much different from the original Buryat cattle. Follow-up efforts could be undertaken to validate the functional effects of the genes/variants revealed in our study using association testing, gene editing, or knockout experiments in livestock or model organisms.

## 5. Conclusions

In this study, we performed a scan for selection signatures in populations of Turano-Mongolian Wagyu and Buryat cattle breeds reared in Russia using whole-genome resequencing data. Our results reveal strong signatures of selection in Wagyu in genes related to meat production traits. In Buryat cattle, we found moderate signatures of selection in genes related to adaptation and feed conversion. The information on several candidate genetic variants reported in this study could be tested to improve genomic selection and marker-assisted selection programs focused on climatic adaptation and meat-related traits in cattle.

## Figures and Tables

**Table 1:** Putative functions for candidate genes detected by >1 window-based method in the Buryat and Wagyu breeds.

Functional Category	Breed	Consensus Interval	Methods	Gene	Source
Cold Climate Adaptation	Buryat	3:33600001-33705000	hapFLK, PBS	*GSTM3*	[54,55]
	Buryat	6:88788570-88838640	hapFLK, PBS	*CXCL8*	[56]
	Buryat	8:72603526-72650000	hapFLK, F*_ST_*	*DOCK5*	[57]
	Buryat	19:42420001-42490000	hapFLK, PBS, DCMS	*STAT3*	[58,59,60,61]
	Wagyu	1:109350001-109425000	hapFLK, F*_ST_*	*RSRC1*	[62]
	Wagyu	16:35455001-35525000	PBS, F*_ST_*	*RGS7*	[63]
	Wagyu	16:63490001-63560000	hapFLK, PBS	*RGSL1*	[64]
Feed Efficiency Traits and Metabolism	Buryat	1:102340001-102410000	hapFLK, PBS	*SI*	[65,66]
	Wagyu	1:109350001-109425000	hapFLK, F*_ST_*	*SHOX2*	[67,68]
	Wagyu	2:103200001-103258854	hapFLK, F*_ST_*	*SNORA70*	[69]
Growth and Development	Wagyu	1:109350001-109425000	hapFLK, F*_ST_*	*SHOX2*	[70,71,72]
	Wagyu	2:90265001-90335000	hapFLK, PBS	*ALS2*	[73]
	Wagyu	2:93978974-94156953	hapFLK, DCMS	*NRP2*	[74]
	Wagyu	2:103200001-103258854	hapFLK, F*_ST_*	*ABCA12*	[75,76]
	Wagyu	3:92470001-92610000	hapFLK, PBS	*GLIS1*	[77,78]
	Wagyu	5:29260001-29325368	hapFLK, PBS	*DIP2B*	[79]
	Wagyu	6:115957434-116027698	hapFLK, PBS	*TNIP2*	[80,81]
	Wagyu	6:115957434-116027698	hapFLK, PBS	*FAM193A* *	[81]
	Wagyu	28:29100001-29119970	hapFLK, F*_ST_*	*P4HA1*	[82]
Immunity and Resistance to Pathogens	Buryat	3:12800001-12845000	PBS, F*_ST_*	*FCRL5*	[83,84,85]
	Buryat	3:33600001-33705000	hapFLK, PBS	*EPS8L3*	[86]
	Buryat	6:88788570-88838640	hapFLK, PBS	*CXCL8*	[87]
	Buryat	19:42420001-42490000	hapFLK, PBS, DCMS	*STAT3*	[88,89]
	Wagyu	5:102242758-102367026	hapFLK, PBS	*WC1-12*	[90,91,92]
	Wagyu	20:23975001-24115000	hapFLK, PBS	*CDC20B*	[93]
	Wagyu	20:23975001-24115000	hapFLK, PBS	*GZMA*	[93,94]
	Wagyu	20:23975001-24115000	hapFLK, PBS	*GZMK*	[93,94]
	Wagyu	20:23975001-24115000	hapFLK, PBS	*ESM1*	[93,95]
Meat Quality Traits	Buryat	6:77455001-77560000	hapFLK, PBS	*ADGRL3*	[96]
	Wagyu	1:109350001-109425000	hapFLK, F*_ST_*	*RSRC1*	[97]
	Wagyu	5:29260001-29325368	hapFLK, PBS	*DIP2B*	[98]
	Wagyu	5:102242758-102367026	hapFLK, PBS	*WC1-12*	[99]
	Wagyu	11:38600001-38675000	PBS, F*_ST_*	*BTA-MIR-216B*	[100]
Milk traits	Buryat	6:77455001-77560000	hapFLK, PBS	*ADGRL3*	[101]
Reproduction	Buryat	3:33600001-33705000	hapFLK, PBS	*GSTM3*	[102]
	Buryat	6:88788570-88838640	hapFLK, PBS	*CXCL8*	[87]
	Buryat	15:51651871-51696907	hapFLK, PBS	*NUMA1*	[103,104]
	Wagyu	1:109350001-109425000	hapFLK, F*_ST_*	*RSRC1*	[105]
	Wagyu	2:90090001-90230000	hapFLK, PBS	*C2CD6*	[106]
	Wagyu	2:90265001-90335000	hapFLK, PBS	*ALS2*	[73]
	Wagyu	5:29260001-29325368	hapFLK, PBS	*DIP2B*	[107]
	Wagyu	11:38600001-38675000	PBS, F*_ST_*	*BTA-MIR-217*	[108]
	Wagyu	11:38600001-38675000	PBS, F*_ST_*	*BTA-MIR-216A*	[109]
	Wagyu	11:38600001-38675000	PBS, F*_ST_*	*BTA-MIR-216B*	[110]

* A missense variant from this gene was detected by single-point F*_ST_* analysis.

## Data Availability

The raw sequencing data for Wagyu and Buryat animals are available from NCBI SRA under the BioProject accession numbers PRJNA1105472 and PRJNA1101073.

## Supplementary Materials

The following supporting information can be downloaded at: https://www.mdpi.com/article/10.3390/ani14142059/s1, File S1: Illumina adapters used for read trimming; Table S1: SRA IDs for animals used in this study; Table S2: Consensus selection signature regions detected by four window-based methods in Wagyu and Buryat breeds and corresponding genes; Table S3: Candidate SNPs from regions putatively selected in Wagyu cattle and their alternative allele frequencies and genotype distributions in the four-breed test set and in the 1000 Bull Genomes Project (1KBGP); Table S4: Candidate SNPs from regions putatively selected in Buryat cattle and their alternative allele frequencies and genotype distributions in the four-breed test set and in the 1000 Bull Genomes Project (1KBGP); Table S5: Candidate SNPs from top 1% of results of single-point F*_ST_* analysis for Wagyu cattle and their alternative allele frequencies and genotype distributions in the 1000 Bull Genomes Project (1KBGP); Table S6: Candidate SNPs from top of 1% results of single-point F*_ST_* analysis for Buryat cattle and their alternative allele frequencies and genotype distributions in the 1000 Bull Genomes Project (1KBGP); Table S7: Functional enrichment analysis for genes detected by four window-based methods and single-point F*_ST_* in Wagyu and Buryat cattle; Table S8: Functional annotation clustering for genes detected by four window-based methods and single-point F*_ST_* in Wagyu and Buryat cattle.
