# Supplementary material for: Scans for Signatures of Selection in Genomes of Wagyu and Buryat Cattle Breeds Reveal Candidate Genes and Genetic Variants for Adaptive Phenotypes and Production Traits
Source: Animals (Basel). 2024 Jul 13;14(14):2059. doi: 10.3390/ani14142059 (PMC11274160; doi:10.3390/ani14142059)
Supplement: Supplementary file 1 [file animals-14-02059-s001.zip › File S1.pdf]

```
>adapter1
AATGATACGGCGACCACCGAGATCTACACTCTTTCCCTACACGACGCTCTTCCGATCT
>adapter2
GATCGGAAGAGCACACGTCTGAACTCCAGTCACATCACGATCTCGTATGCCGTCTTCTGCTTG
```
